# Reversing the Trend of Childhood Obesity

**Published:** 2009-06-15

**Authors:** Donna F. Stroup, Valerie R. Johnson, Robert A. Hahn, Dwayne C. Proctor

**Affiliations:** Data for Solutions, Inc; Centers for Disease Control and Prevention, Atlanta, Georgia; Centers for Disease Control and Prevention, Atlanta, Georgia; Robert Wood Johnson Foundation, Princeton, New Jersey

## Introduction

In March 2008, a group of experts in anthropology, law, epidemiology, ethics, and social networking met to share their diverse perspectives on preventing childhood obesity. In meeting their charge to identify innovative ways to lower the prevalence of childhood obesity, they asked several questions: What has succeeded and what has not? What are the barriers to success? Whose job is it to address these barriers? We provide a brief background on childhood obesity and highlight some of the ideas generated at the Symposium on Epidemiologic, Ethical, and Anthropologic Issues in Childhood Overweight and Obesity, which took place in March 2008 at Saint George's University, Saint George, Grenada.

## Major Global Health Problem of Our Time

Childhood obesity and overweight are well recognized as a major health problem in the United States, particularly in poor and underserved populations (1). The proportion of children who are overweight or obese has been increasing since the mid-1970s (2). The problem gained widespread attention in 2001, when then US Surgeon General David Satcher issued a Call to Action to tackle what he declared to be a "public health crisis" (3). In response, the Institute of Medicine (IOM) launched a plan to decrease the prevalence of overweight and obesity among children and youth in the United States (4).

Childhood obesity is a problem not just in the United States. It is now a problem in nearly all developing regions of the world. An estimated 155 million school-aged children around the world are overweight or obese, according to the International Obesity Taskforce (5). The prevalence of childhood obesity is growing rapidly even in countries where hunger is also a problem (6,7). Even more alarming is that risk factors for diabetes and heart disease — long considered to be diseases of adulthood — are now affecting children (8,9). These obesity-related diseases are being diagnosed at earlier ages among children (10), making childhood obesity "the greatest single global public health problem of our time," according to Sir Kenneth Stuart, MD (oral communication, International Medical Education Trust, March 2008). Poor eating habits and insufficient physical activity, behaviors that are learned in childhood, are major contributors to obesity and its resulting health problems (11).

Mathers and Loncar (12) updated projections from the Global Burden of Disease project (13) and predicted that the proportion of deaths caused by noncommunicable diseases such as diabetes and heart disease could rise from 59% in 2002 to 69% in 2030. These could be conservative estimates if economic growth in low-income countries is lower than the forecasts used in these projections. Thus, future trends in unhealthy behaviors linked to childhood obesity will play a key role in determining whether these serious health problems are reversed.

In planning ways to reduce the prevalence of obesity, we have much to learn from our past. In the United States, for example, diverse cultures, attitudes about foods and what constitutes an appropriate body size, major changes in food production, and reduced opportunities for physical activity have worked together to create an environment where childhood obesity has flourished. Research has taught us that no single approach will work. To effectively address childhood obesity, we must engage diverse disciplines and partners, especially people in the communities that are hurt the most by childhood obesity. Thus, in the United States, the Robert Wood Johnson Foundation has focused on strategies to combat childhood obesity in schools and communities (Figure). The foundation supports 5 key strategies:

- Increase the availability of healthy foods at home.
- Offer healthy food choices at schools.
- Increase physical activity in schools.
- Increase physical activity in communities.
- Reduce children's screen time at home.

Whether these approaches — alone or in combination — are actually making a difference remains to be determined. The reasons are complex.

**Figure 1:**
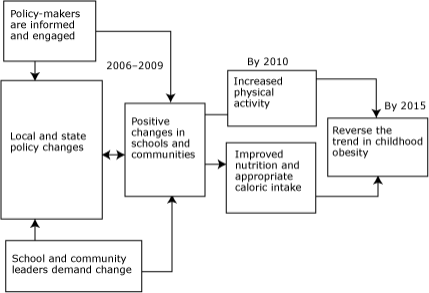
Abbreviated logic model for childhood obesity interventions, The Robert Wood Johnson Foundation

## Barriers to Childhood Obesity Interventions

First, we have limited evidence to know what interventions will work in targeting childhood obesity. The Task Force on Community Preventive Services, an independent expert group funded by the US government, is responsible for systematically determining which preventive programs have been scientifically shown to benefit communities. Task Force findings for obesity and physical activity are published in the *Guide to Community Preventive Services* (www.thecommunityguide.org). The Task Force has reviewed 25 community-level obesity interventions for children and adults. It recommended 10 of these and concluded that the remaining 15 are not yet backed by enough evidence to conclude that they work. Interventions discussed in this issue of *Preventing Chronic Disease* address not only obesity but also diet, physical activity, and the built environment. All 10 of the recommended interventions focus on increasing physical activity, although some are work-site interventions not relevant to this discussion (14) (Table). The IOM recognized this limitation on evidence in its 2005 report but nonetheless decided to support actions "based on the best available evidence — as opposed to waiting for the possible evidence."

Second, even when scientists identify interventions that are known to work in some settings, we must consider the barriers and the resistance we might encounter in other settings. For example, an intervention designed to be delivered in schools may be 100% effective in the study settings. But what if only 50% of schools in the United States are willing to conduct the program, and in those willing schools, only 25% of teachers are willing to incorporate the intervention into lesson plans, and in the classes that do use this highly effective intervention only 50% of students pay attention when the intervention is presented, only half of whom follow through on the intervention on their own time? That 100% effective intervention now benefits only 3% of the children. Why? What happened? Clearly, issues beyond classic epidemiology can lower the impact that childhood obesity interventions might have. This view is echoed in a recent report from the Trust for America's Health and the Robert Wood Johnson Foundation, which concludes that although many promising policies have been developed to promote physical activity and nutrition in US communities, these policies are not being adopted or practiced at levels needed to reverse obesity trends (15).

A third barrier to the successful implementation of childhood obesity interventions is the lack of agreement about whether childhood overweight is an individual (family) or societal issue. A 2006 poll conducted by Research!America and the Endocrine Society showed that 27% of adults in the United States consider overweight and obesity to be a top health issue for children; just over half of respondents said that the problem is a public health issue that society has a responsibility to address, whereas 46% said it is a private issue that individuals should address (16).

Finally, even strategies that are successful for children, their families, and their communities in the United States are unlikely to be successful in slowing the global acceleration of this pandemic. Many strategies may need to be country-specific because of the unique political, economic, and social influences that affect diet and physical activitiy in diverse cultures. Recognizing the need for health-promoting school educational reforms, the World Health Organization is collaborating with the European Commission and the Council for Europe to design a strategy for improving school-aged children's early years by helping them to develop healthy lifestyles. Five hundred schools in 40 countries in Europe have become members of the European Network of Health Promoting Schools (17).

The articles in this collection explore these challenges facing childhood obesity interventions. The analyses and recommendations come from a panel of experts who addressed interdisciplinary issues inherent in societal approaches to childhood obesity.

Samantha Graff and Jacob Ackerman (Public Health Law and Policy Institute) draw from the California tobacco control experience to illustrate the role of public health laws in promoting public health and how the strategies used in California can be applied to childhood obesity prevention (18). They discuss laws pertaining to taxation, land use, product sales, and consumption; explore which legislative and regulatory tactics of tobacco control may apply to childhood obesity prevention; and discuss where the differences between the products, conduct, and consumers at stake may call for new tactics.

Social and community approaches to halting the increase in childhood obesity prevalence are important, but recent work has shown that obese youth might also benefit from individualized interventions (19). Laura Koehly and Aunchalee Loscalzo (National Human Genome Institute, National Institutes of Health) explore the contribution of social relationships to the development of obesity through the interaction of genetic, behavioral, and environmental factors (20). Using social network structure, communities could be more successful in motivating physical activity among children and their families.

Keshia Pollack (Johns Hopkins University) emphasizes the need to attend to injury prevention as we develop interventions to encourage physical activity (21). Her recommendations could enhance safe environments where children and families can be physically active with lower risk of injury. Frank Chaloupka and Lisa Powell (Institute for Health Research and Policy, University of Illinois at Chicago) provide new evidence on the importance of food pricing and availability of food stores and restaurants in relation to active living and weight outcomes among youth (22). Patricia Thompson-Reid (Centers for Disease Control and Prevention) describes a framework for engaging communities to increase their awareness of childhood obesity as a public health problem and a commitment to a process for developing solutions (23).

Recommendations from these fresh perspectives are intended to help communities be effective in addressing childhood overweight by encouraging healthy, active lifestyles among youth. Taken together, these analyses offer insight into how we might tackle childhood obesity and offer children the promise of a healthy future, free of obesity and the host of health problems that it brings.

## Figures and Tables

**Table.** Findings for Interventions That Target Obesity, *Guide to Community Preventive Services* a

**Program and Setting**	**Number of Programs Reviewed**	**Number of Programs Recommended**
Individual/home-based programs	2	1
School/college-based programs	7	1
Worksite-based programs	2	2
Community-based programs	8	6
Health care-based programs	6	0
Total	25	10

a The Task Force recommended 10 of the interventions it reviewed and concluded that, for the remaining 15 interventions, insufficient evidence exists to recommend them definitively. All 10 of the recommended interventions are directed at increasing physical activity, although some are worksite interventions not relevant to a discussion of childhood obesity. Interventions that were reviewed address not only obesity but also diet, physical activity, and the physical environment.
